# Selected Thoughts on Hydrophobicity in Drug Design

**DOI:** 10.3390/molecules26040875

**Published:** 2021-02-07

**Authors:** Lillian L. Lou, John C. Martin

**Affiliations:** Nexus Development PA, Redwood City, CA 94065, USA; lillianlou@nexusdevpa.com

**Keywords:** hydrophobic interaction, drug design, acyclic nucleoside phosphonates, nucleotide antivirals

## Abstract

The fundamental aim of drug design in research and development is to invent molecules with selective affinity towards desired disease-associated targets. At the atomic loci of binding surfaces, systematic structural variations can define affinities between drug candidates and biomolecules, and thereby guide the optimization of safety, efficacy and pharmacologic properties. Hydrophobic interaction between biomolecules and drugs is integral to binding affinity and specificity. Examples of antiviral drug discovery are discussed.

## 1. Introduction

We are pleased to contribute to a collection of manuscripts dedicated to Professor Erik De Clercq on the occasion of his 80th birthday. One of us (J.C.M.) has a four-decade collaboration with Professor De Clercq dating back to our early and independent work on potential antiviral drugs since I synthesized ganciclovir [1,2]. My career received a substantial boost as one of four plenary speakers at an American Chemical Society Symposium at the ACS National Meeting in 1983 in Seattle. Erik and I were the younger up-and-coming scientists, discussing BVDU (bromovinyldeoxyuridine) [3] and ganciclovir (Cytovene^®^) [4], respectively. The more established scientists were Roland Robins (ribavirin, Virazole^®^) [5] and Jack Fox (2′-fluoro-arabinosyl pyrimidine nucleosides) [6]. Erik and I were already friends; however, that symposium began a long series of frequent meetings at conferences around the world to explore our common scientific interests, especially the chemistry of antivirals. The decade of the 1980s was a great start to our collaboration, subsequent to our independent publication of the first examples of antiviral nucleotide analogues [7,8]. The formal collaboration was initiated in 1986 along with Professor Antonín (Tony) Holý in Prague to explore the potential of phosphonomethyl nucleotide analogues as antiviral drug candidates. Just two years into the collaboration, I presided over a full day the ACS Symposium (Los Angeles, CA, USA, 1988), covering the then newly discovered antiviral potential of nucleotide analogues. New results and concepts of this emerging field were captured in a book, Nucleotide Analogues as Antiviral Agents, edited by me, where 12 chapters were contributed by a number of pioneering labs including Erik and Tony (Chapter 4) [9]. Along the way we collaborated on other endeavors including the anti-HIV drug stavudine (Zerit^®^) [10], and shared many a stage as experts in the field, most recently as guests of Jean-Marie Lehn at the University of Strasbourg, 23 September 2019.

The following covers some brief observations of drug discovery concerning the power of lipophilic interactions in drug design and for nucleotide analogues.

## 2. Hydrophobicity Matters

The value of hydrophobic interactions to improve inhibitor affinity and selectivity in drug design has been well recognized and plays out over and over in a variety of research efforts. Even the addition of a methyl group can be profound [11,12,13]. A nucleobase modification discovered at Gilead Sciences was the higher affinity of 5-propynyl substituted pyrimidine antisense oligonucleotide analogues. It was previously known that a 5-methyl-C substitution led to higher affinity binding of oligonucleotides to RNA. The rational assumption by Brian Froehler that creating more hydrophobic stacking by occupying more available space with the linear propyne group proved accurate [14,15].

Around the same time at Gilead, a much more impactful observation was made by serendipity. Choung Kim’s practice of not making assumptions about possible biological activity routinely assayed his chemical intermediates. By this means, oseltamivir (Tamiflu^®^) for influenza virus infection was discovered when a hydrophobic pocket at the virus neuraminidase active site was uncovered then exploited, greatly improving affinity through hydrophobic interactions thus achieving potent inhibition of the influenza virus [16,17].

These lessons have been applied with various success but to no greater degree than subsequent research on nucleotides, discussed below.

## 3. Antiviral Nucleotide Analogues

During the 1970s, Erik worked to establish a strong antiviral research team at the Rega Institute in Leuven, Belgium. By the 1980s, this institute became a favorite collaborator for chemists all over the world seeking to discover new antiviral agents. Foremost of those was Tony Holý at the Institute of Organic and Biochemistry in Prague. Tony was the type of scientist that was never discouraged by negative results and was happy to rely on both hypothesis and serendipity to create important novel observations. For instance, most chemists did not work on nucleotide analogues after an early report that the phosphonate nucleotide analogue of adenosine monophosphate (AMP) was devoid of biological activity [18]. The conclusion at the time was that the polar nature of a phosphonate prevented the molecule from transversing the cell membrane. Tony ignored this precedent and used diethylphosponomethyl tosyalte to O-alkylate a number of nucleoside analogues to see if the resulting nucleotides would have biological activity. Two, HPMPC ((*S*)-1-(3-hydroxy-2-phosphonylmethoxypropyl)cytosine) and PMEA (9-(2-phosphonylmethoxyethyl)adenine), were the subject of a 1986 *Nature* publication that even made the prescient prediction that this class might find use for the treatment of HIV infection [7].

As with many predictions, this one only delivered many years later with much effort by a large group of dedicated scientists. The first approved nucleotide HPMPC (cidofovir, Vistide^®^) only made it to the market 10 years later and had a very limited cytome galovirus retinitis indication. For PMEA, there was great hope for HIV/AIDS but it showed cumulative renal toxicity longer term. Because of the narrow therapeutic window, PMEA’s prodrug adefovir dipivoxil (Hepsera^®^) [19] at a much lower dose than tested for HIV made it to the market for the treatment of hepatitis B infection.

**Figure 1:**
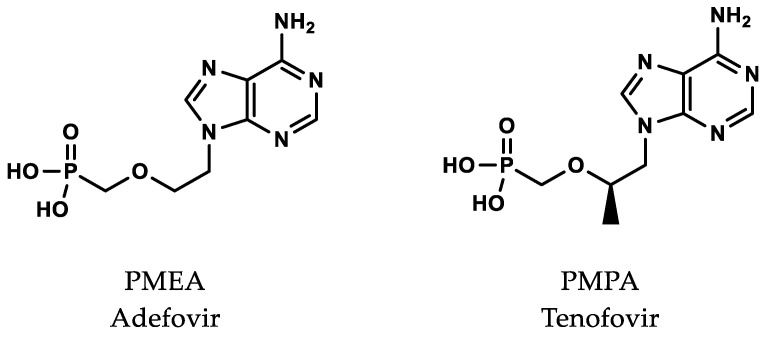
PMEA and PMPA differ in structure by one methyl group.

A large library of nucleotide analogues existed, among which PMEG (9-(2-phophonylmethoxyethyl)guanine) is the most potent and toxic of this new class of molecules. The simple exercise was to probe hydrophobic faces to find higher affinity and better selectivity by substituting with methyl groups in specific locations about the molecule. This effort could result in a new lead, or even a drug candidate. (*R*)-2′-methyl-PMEG looked very promising in that the selectivity was improved in cells [20]. However, animal studies still showed unacceptable toxicities. A similar exercise with the less potent PMEA was much more fruitful. (*R*)-2′-methyl-PMEA was found to be a highly selective inhibitor against HIV [21]. This molecule is known by the acronym PMPA ((*R*)-9-(2-phosphonylmethoxypropyl)adenine) (Figure 1). Its diphosphorylated metabolite was shown to have little affinity for the human host polymerases, especially mitochondrial DNA polymerase γ [22,23].

Hydrophobic probing of these nucleotides by systematic methyl substitutions taught that, while PMEA and PMPA have comparable antiviral activities in vitro [19,20,21,24,25] and the corresponding diphosphates have comparable inhibition against HIV reverse transcriptase [23], PMEA diphosphate is highly efficient and PMPA diphosphate is not, in becoming incorporated into human mitochondrial DNA by DNA polymerase γ [22]. This remarkable difference (by 60-fold) in interfering with host cell metabolism might explain, in part, the differential toxicity profiles between PMEA and PMPA.

PMPA later, developed as prodrugs, led to the transformation of HIV treatment, becoming the mainstay therapy for patients around the world.

## 4. Prodrugs

**Figure 2:**
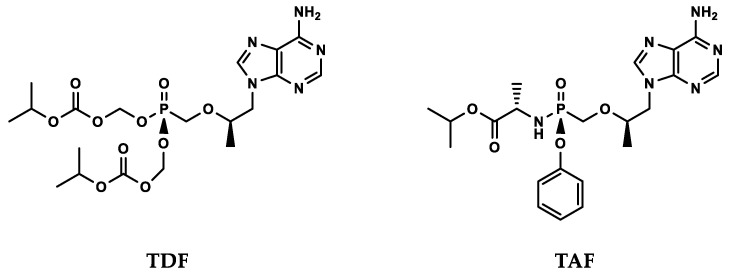
Two prodrugs of tenofovir—once daily oral dosing. TDF: tenofovir disoproxil fumarate; TAF: tenofovir alafenamide.

To make the nucleotide molecules drug-like, prodrugs were employed to allow oral dosing and improved pharmacokinetics. After considerable additional effort, two prodrugs of PMPA (tenofovir) were successfully developed. Tenofovir disoproxil fumarate (TDF 300 mg, Viread^®^) was approved in 2001 for HIV/AIDS and in 2008 for chronic hepatitis B, and tenofovir alafenamide (TAF 40 mg, Vemlidy^®^) was approved in 2016 (Figure 2).

Modification of tenofovir by prodrug moieties markedly increased uptake into cells, as TDF is 50-fold and TAF several hundred- to one thousand-fold more active in vitro than tenofovir [25,26,27]. As the first prodrug in development, TDF’s clinical potency is limited by the rapid hydrolysis to tenofovir in blood, resulting in suboptimal levels of TDF in circulation. The second prodrug TAF has much improved stability in circulation, efficiency in cellular uptake and selective proteolytic conversion to tenofovir inside specific target cells [26,28]. Consequently, TAF can achieve equivalent clinical antiviral efficacy as TDF when dose is lowered by 8- to 10-fold [29,30]. The resulting decrease in off-target effects renders an improved safety profile [31].

Both TDF and TAF were subsequently combined with other agents to produce single tablet regimens, which transformed the care of HIV patients. The various formations are shown in Table 1. The later developed formulations containing TAF have supplanted the earlier TDF combination products. This is because TAF has a superior safety profile which is important for patients with HIV and HBV [32] who are being individually treated for decades.

## 5. Concluding Remarks

The collaboration with Erik in Belgium and Tony in the Czech Republic has spanned 35 years and involved hundreds of scientists working together to achieve remarkable benefits for patients, allowing many to live normal productive lives instead of succumbing to fatal disease. Starting about 15 years ago, global efforts brought tenofovir-containing regimens to low-income countries, greatly expanding the benefits of tenofovir around the world. This collaboration, which has paralleled the long careers of many of our colleagues, demonstrates Erik’s contribution to have created many scientific progeny who carry on important research in virology to this day, including current efforts to control COVID-19, the disease caused by SARS-CoV-2.

## Figures and Tables

**Table 1:** Tenofovir-containing therapeutics for HIV and HBV.

Disease	Drug (Common Name)	Launch
HIV/AIDS	Viread (TDF)	2001
	Truvada^®^ (TDF/emtricitabine)	2004
	Atripla^®^ (TDF/emtricitabine/efavirenz) ^1^	2006
	Complera^®^ (TDF/emtricitabine/rilpivirine) ^1^	2011
	Stribild^®^ (TDF/emtricitabine/elvitegravir/cobicistat) ^1^	2012
	Genvoya^®^ (TAF/emtricitabine/elvitegravir/cobicistat) ^1^	2015
	Odefsey^®^ (TAF/emtricitabine/rilpivirine) ^1^	2016
	Descovy^®^ (TAF/emtricitabine)	2016
	Bitarvy^®^ (TAF/emtricitabine/bictegravir) ^1^	2018
HIV PrEP ^2^	Truvada (TDF/emtricitabine)	2012
	Descovy (TAF/emtricitabine)	2019
Hepatitis B	Hepsera (adefovir dipivoxil)	2002
	Viread (TDF)	2008
	Vemlidy (TAF)	2016

^1^ Single tablet regimens dosed as one pill once daily; ^2^ pre-exposure prophylaxis for the prevention of HIV transmission.

## Data Availability

All data described are from published reports as referenced and are publicly accessible.
